# Supplementary material for: QTL Mapping for Age-Related Eye Pigmentation in the Pink-Eyed Dilution Castaneus Mutant Mouse
Source: Genes (Basel). 2022 Jun 24;13(7):1138. doi: 10.3390/genes13071138 (PMC9318509; doi:10.3390/genes13071138)
Supplement: Supplementary file 1 [file genes-13-01138-s001.zip › Supplementary Figures.pdf]

Supplementary Figures

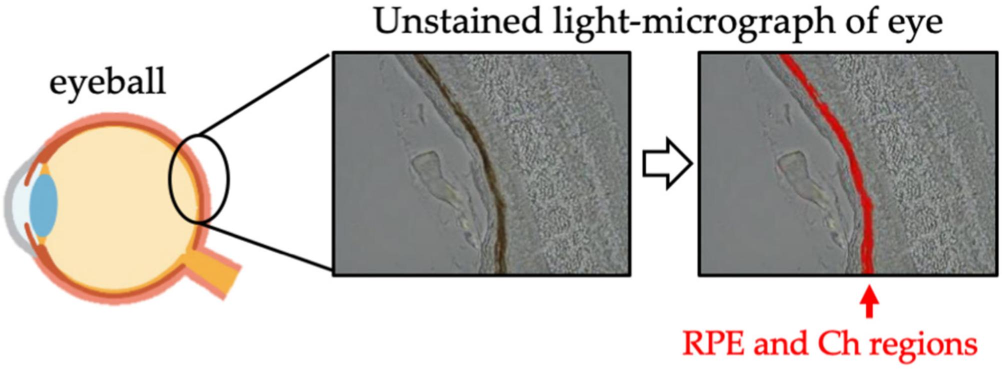

Figure S1. Measurement site of eye pigmentation. The brightness of pigmentation in both regions of retinal pigment epithelium (RPE) and choroid (Ch) was measured by image analysis (see Materials and Methods for details).

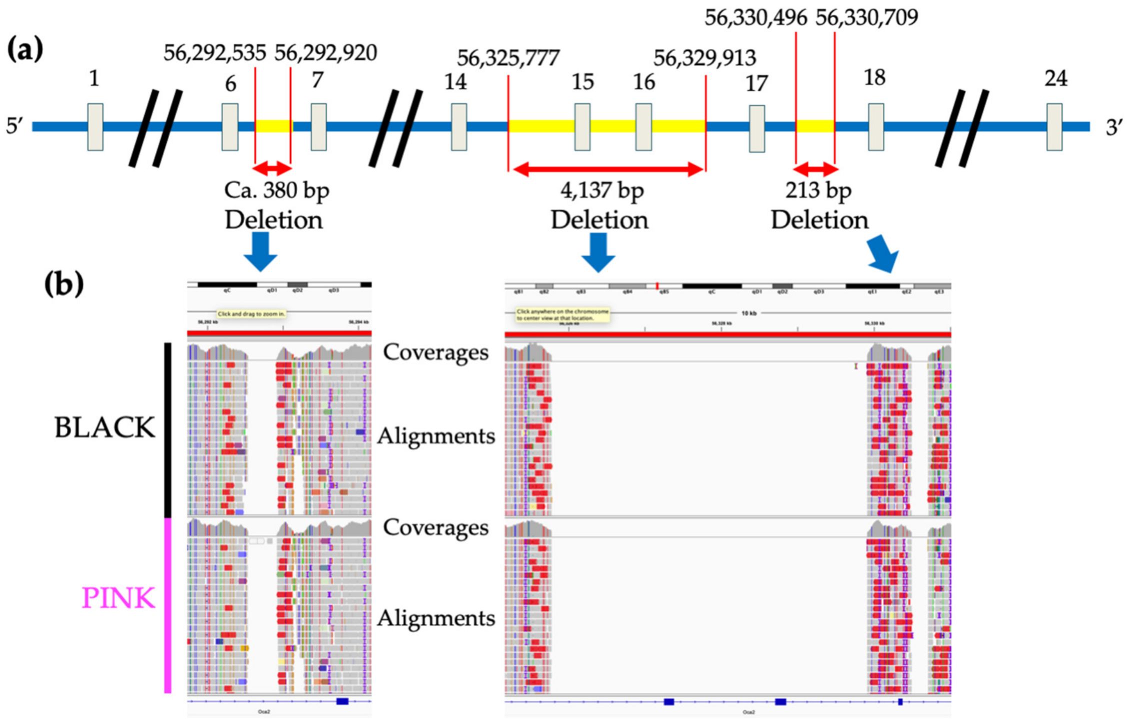

Figure S2. Sequence analysis of the *Oca2* gene for PINK and BLACK mice. (a) Schematic of the nucleotide sequence; (b) Split-screen view of read alignments for deletion regions from the two mice obtained by IGV software. The numbers on boxes represent exon numbers; Red double-headed horizontal arrows show deleted regions. The numbers on the red vertical lines represent the physical map positions (bp) of the borders of the deletion.

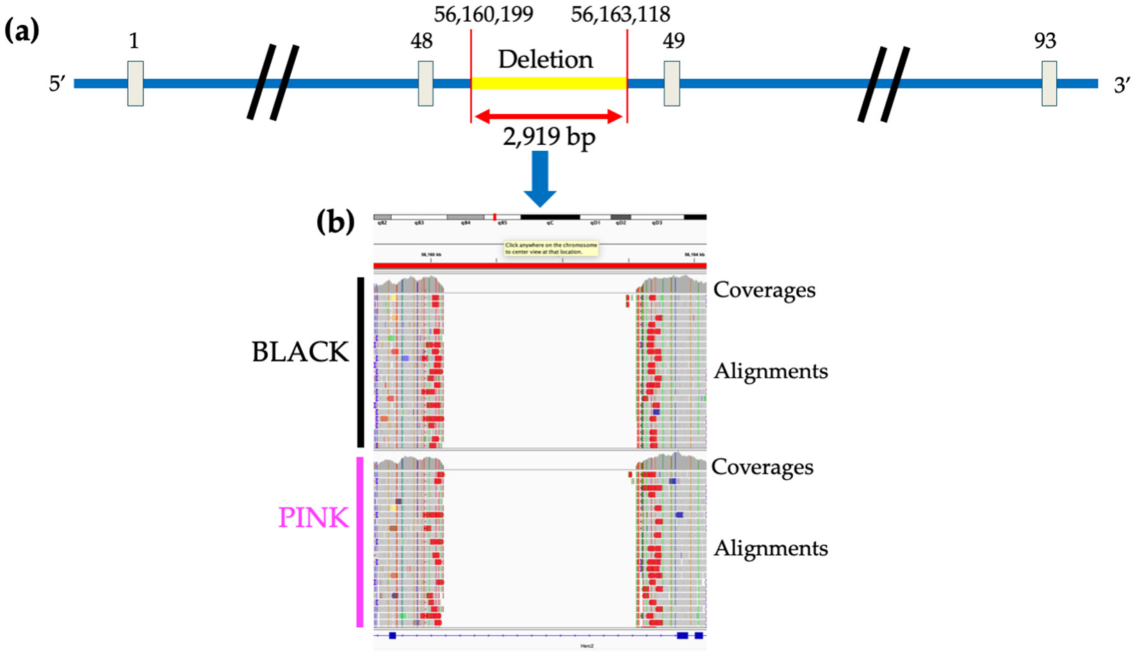

Figure S3. Sequence analysis of the *Herc2* gene for PINK and BLACK mice. **(a)** Schematic of the nucleotide sequence; **(b)** Split-screen view of read alignments for deletion regions from the two mice obtained by IGV software. The numbers on boxes represent exon numbers; The red double-headed horizontal arrow shows a deleted region. The numbers on the red vertical lines represent the physical map positions (bp) of the borders of the deletion.

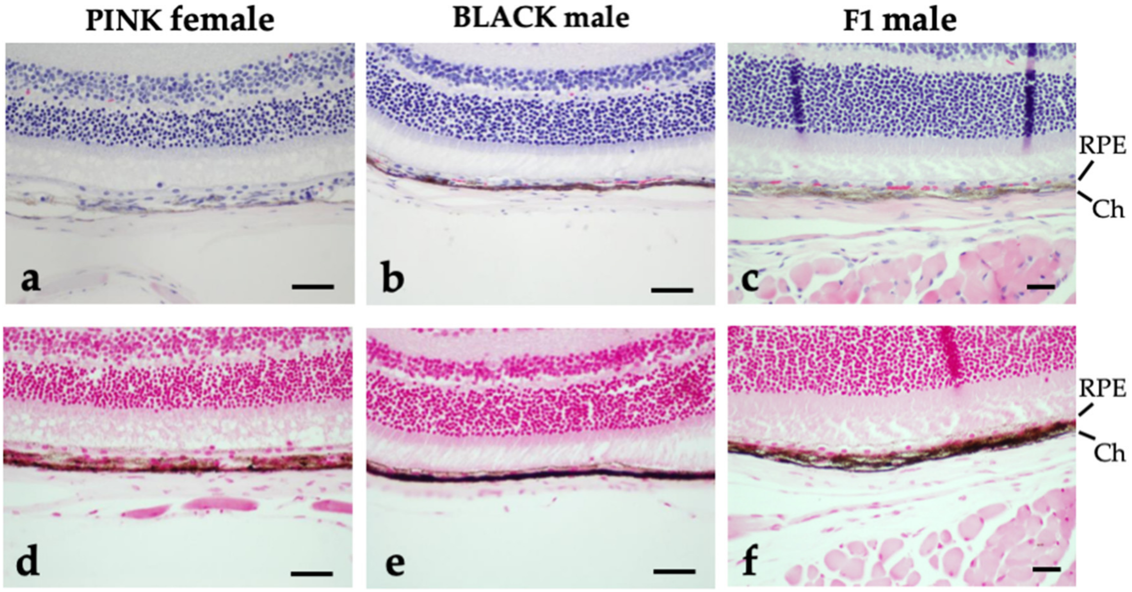

Figure S4. Light microscopic analysis of eyes from PINK and BLACK parental strains at 9-11 months of age and their F1 hybrid at 4 months of age. **(a-c)** Hematoxylin-eosin strained micrographs; **(d-f)** Fontana-Masson stained micrographs. RPE and Ch indicate retinal pigment epithelium and choroid, respectively. Scale bars show **(a,b,d,e)** 50 μm and **(c,f)** 20 μm.

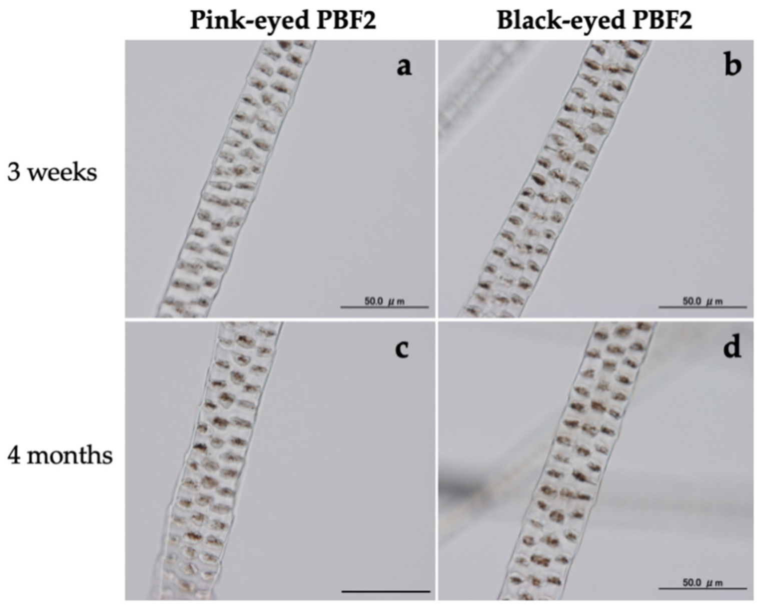

Figure S5. Age-related changes in color of awl hairs obtained from the dorsal regions of pink-eyed and black-eyed PBF2 mice. **(a,b)** Light micrographs at 3 weeks of age; **(c,d)** Light micrographs at 4 months of age. Scale bars show 50 μm.

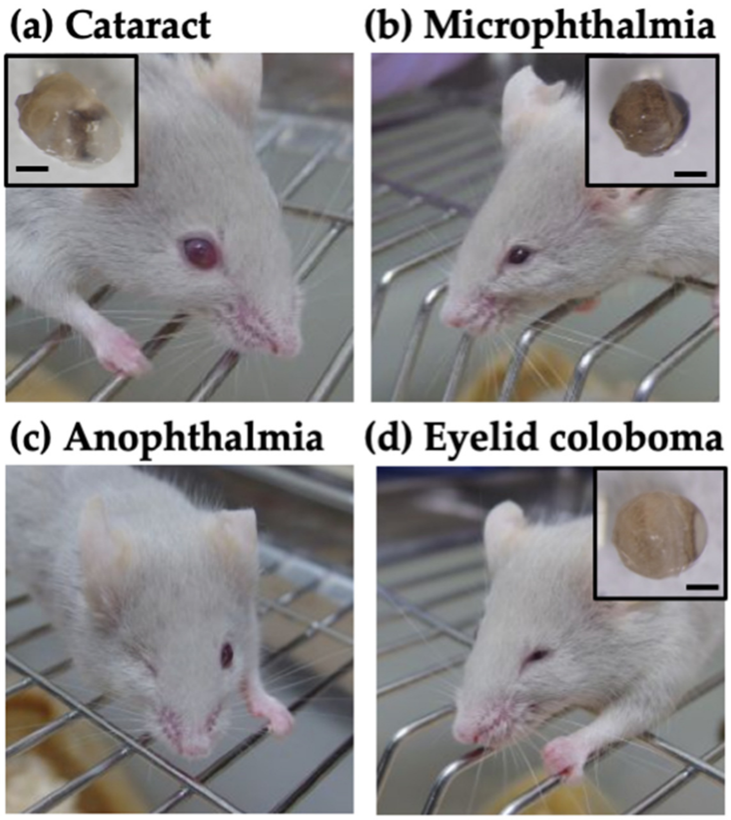

Figure S6. External appearances of mice with eye abnormalities at 4 months of age found in the PBF2 population. **(a)** Cataract; **(b)** Microphthalmia; **(c)** Anophthalmia; **(d)** Eyelid coloboma. Insets indicate stereomicrographs of the eye abnormalities. Scale bars show 1 mm.

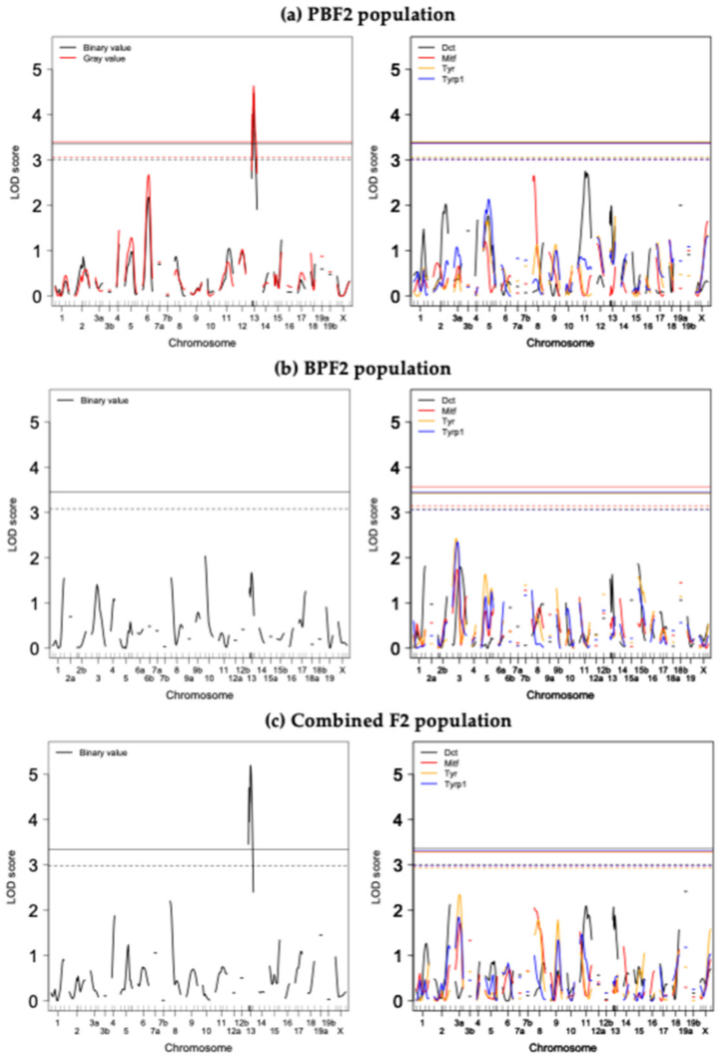

Figure S7. Genome-wide LOD score plots for traits of binary values, gray values and gene expression levels (*Dct*, *Mitf*, *Tyr* and *Tyrp1*). **(a)** PBF2 population; **(b)** BPF2 population; **(c)** Combined F2 population. Solid and dashed horizontal lines show genome-wide 5% and 10% significant threshold levels obtained by 10,000 permutation tests for each trait (see Table S8). The small vertical line on the x-axis indicates the position of the DNA marker.

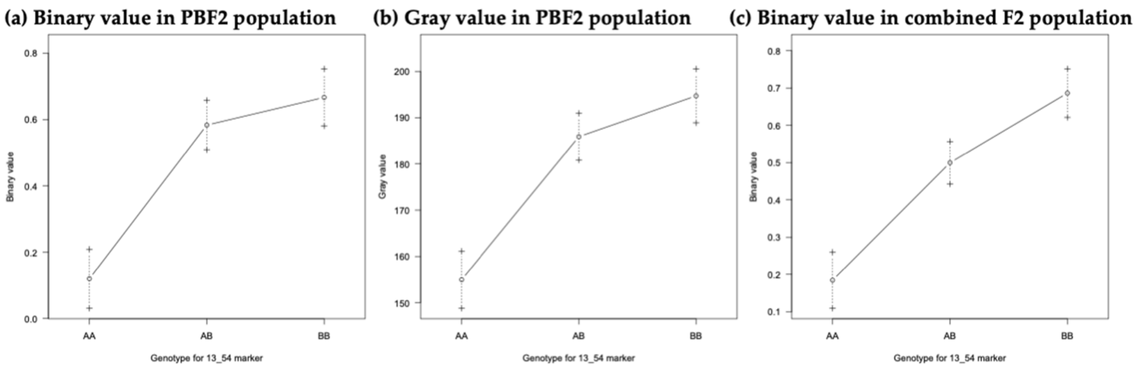

Figure S8. Effect plots of QTLs for binary and gray values on chromosome 13. **(a,b)** PBF2 population; **(c)** Combined F2 population.

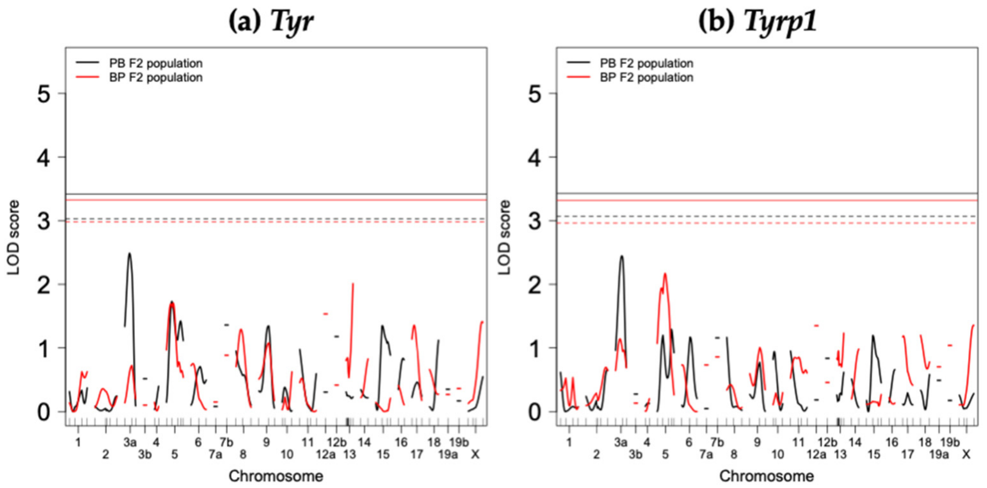

Figure S9. Genome-wide LOD score plots by population for *Tyr* and *Tyrp1* expression levels that showed a significant difference between PBF2 and BPF2 populations (see Table 2). **(a)** *Tyr* expression; **(b)** *Tyrp1* expression. Solid and dashed horizontal lines show genome-wide 5% and 10% significant threshold levels obtained by 10,000 permutation tests for each trait (see Table S9). The small vertical line on the x-axis indicates the position of the DNA marker.

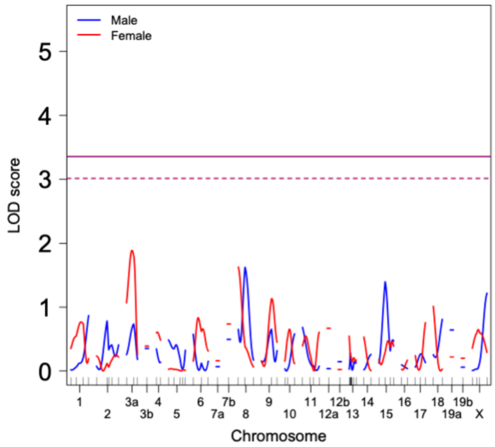

Figure S10. Genome-wide LOD score plots by sex for *Tyr* expression levels that showed a significant difference between male and female mice (see Table 2). Solid and dashed horizontal lines show genome-wide 5% and 10% significant threshold levels obtained by 10,000 permutation tests for each trait (see Table S9). The small vertical line on the x-axis indicates the position of the DNA marker.
